# Comparison of Necroptosis With Apoptosis for OVX-Induced Osteoporosis

**DOI:** 10.3389/fmolb.2021.790613

**Published:** 2021-12-24

**Authors:** Bin He, Yongjun Zhu, Hongwang Cui, Bo Sun, Tian Su, Peng Wen

**Affiliations:** ^1^ Department of Spine and Osteopathic Surgery, The First Affiliated Hospital of Hainan Medical University, Haikou, China; ^2^ Department of Orthopedics, The First Affiliated Hospital of Chongqing Medical University, Chongqing, China; ^3^ Department of Nephrology, The First Affiliated Hospital of Hainan Medical University, Haikou, China

**Keywords:** necroptosis, apoptosis, ovariectomy, RIPK3, *in vivo*, *in vitro*, osteocytes

## Abstract

As one common kind of osteoporosis, postmenopausal osteoporosis (PMOP) is associated with the death and excessive loss of osteocytes. Estrogen deficiency of PMOP can cause osteocyte death by regulating necroptosis and apoptosis, but their roles in POMP have not been compared. In the present study, ovariectomy (OVX)-induced rat and murine long bone osteocyte Y4 (MLO-Y4) cells were used to compare the influence of necroptosis and apoptosis on osteocyte death and bone loss. Benzyloxycarbonyl-Val-Ala-Asp (zVAD) and necrostatin-1 (Nec-1) were used to specifically block cell apoptosis and necroptosis, respectively. OVX rats and MLO-Y4 cells were divided into zVAD group, Nec-1 group, zVAD + Nec-1 group, vehicle, and control group. The tibial plateaus of the rat model were harvested at 8 weeks after OVX and were analyzed by micro–computed tomography, transmission electron microscopy (TEM), the transferase dUTP nick end labeling assay, and western blot. The death of MLO-Y4 was stimulated by TNF-α and was measured by flow cytometry and TEM. The results found that necroptosis and apoptosis were both responsible for the death and excessive loss of osteocytes, as well as bone loss in OVX-induced osteoporosis, and furthermore necroptosis may generate greater impact on the death of osteocytes than apoptosis. Necroptotic death of osteocytes was mainly regulated by the receptor-interacting protein kinase 3 signaling pathway. Collectively, inhibition of necroptosis may produce better efficacy in reducing osteocyte loss than that of apoptosis, and combined blockade of necroptosis and apoptosis provide new insights into preventing and treating PMOP.

## Introduction

Osteoporosis is one systemic skeletal disease characterized by low bone mass and microarchitectural deterioration of bone tissue, which increases susceptibility to fractures (Ensrud and Crandall, 2017; Kanis et al., 2019). With the aging of a population, the incidence of postmenopausal osteoporosis (PMOP) is enormously increased and may lead to serious osteoporotic fractures (Fang et al., 2020; He et al., 2021; Zhao et al., 2021). Osteocytes, osteoclasts, and osteoblasts have important roles in bone remodeling and the incidence of osteoporosis (Han et al., 2018; Robling and Bonewald, 2020; Udagawa et al., 2021). Osteocytes are known as ramified bone cells distributed throughout the bone matrix and subsequently form 90–95% of adult bone cells (Milovanovic et al., 2013; Mattinzoli et al., 2014). Osteocytes play a critical role in transmitting various signals to the surfaces between osteoclasts and osteoblasts (Kalajzic et al., 2013), suggesting that osteocytes may also participate in regulating bone resorption and formation (Heino et al., 2004). Especially, osteocyte death is confirmed to cause PMOP (Dallas et al., 2013).

Increasing evidence indicates that ongoing loss of osteocytes caused by cell death is an important factor that results in PMOP, and osteocyte apoptosis has been reported to be a crucial factor (Cui et al., 2016a; Vakili et al., 2021). Previous studies have demonstrated that apoptosis of osteocytes is accelerated within 4 weeks of estrogen withdrawal (Cardoso et al., 2009; Emerton et al., 2010). In addition to apoptosis, necroptosis is another important approach that causes cell death and is regarded as one subtype of necrosis (Degterev et al., 2014). Necrotic cell death modalities contribute to different pathologies including necroptosis, pyroptosis, and parthanatos (Robinson et al., 2019). Apoptosis is programed cell death with the features of cell shrinkage and nuclear condensation and is regulated by multiple pathways including the autophagy pathway, mitogen-activated protein kinase–dependent antioxidant signaling, overproduction of reactive oxygen species, and WNT/β-catenin signaling (Ru and Wang, 2020). Necroptosis is highly regulated caspase-independent programed cell death with morphological similarities to necrosis, including increased cell volume, organelle swelling, plasma membrane rupture, and subsequent loss of intracellular contents, which are regulated by receptor-interacting protein kinase 1/3 (RIPK1/RIPK3) signaling and can be blocked by necrostatin-1 (Nec-1) intervention (Linkermann et al., 2012; Cui et al., 2016a). Many inflammatory signals such as tumor necrosis factor-α (TNF-α) and Toll-like receptors also participate in the pathological processes of necroptosis (Belizário et al., 2015).

Apoptosis and necroptosis, two different types of cell death, display crucial roles in the loss of osteocyte and bone mass. Estrogen loss in ovariectomy (OVX) animals significantly increases apoptosis and necroptosis of osteocytes, as well as osteoclastic resorption (Emerton et al., 2010; Cui et al., 2016b), which are modulated by many factors including the types of bone tissues, specific compartments, and the time of OVX (Emerton et al., 2010; Gerbaix et al., 2017; Fu et al., 2020). Our previous studies demonstrated that necroptosis is one important approach to cause osteocyte death and subsequent osteoporosis in rats undergoing OVX (Cui et al., 2016a; Cui et al., 2016b). The progressive depletion of osteocytes due to necroptosis promoted the progression of PMOP in OVX rats, and necroptotic death of osteocytes could be blocked by treatment with necrostatin-1 (Nec-1, a specific inhibitor of receptor-interacting protein 1), which subsequently attenuated bone loss (Cui et al., 2016b). However, the roles of necroptosis and apoptosis for osteocyte loss have not been well established, and the aim of the present study is to compare necroptosis with apoptosis in the progressive loss of osteocytes in rats with estrogen deficiency.

## Materials and Methods

### Animals and Grouping

The study protocol was approved by the ethics committee of the First Affiliated Hospital of Hainan Medical University (Haikou, China). All animal experimental procedures were carried out in accordance with the guidelines laid down by the National Institute of Health (United States) and the National Laws and Regulations.

Female Sprague Dawley (SD) rats aged 16 weeks (Animal Laboratory Center of Chongqing Medical University, Chongqing, China) were housed under standard laboratory conditions (12-h light/dark cycle, temperature 22 ± 2°C, and humidity 55 ± 5%) with free access to water and standard rodent diet for a 7-day adaptation period. A total of 62 animals were randomly divided into OVX group (bilateral OVX, n = 50) and control group (sham operation, n = 12). Anesthesia was administered by subperitoneal injection with pentobarbital sodium (30–50 mg/kg). Among the OVX group, two rats died during the bilateral OVX surgery because of deep anesthesia. For the sham operation in the control group, the ovaries were exteriorized and then replaced back in the abdominal cavity. We observed the postoperative recovery, diet, wound healing, and daily activities after OVX. All rats were housed under standard laboratory conditions with free access to water and a standard rodent diet. All of them gradually obtained complete recovery and good wound healing.

### Drug Administration

Drugs included necrostatin-1 (Nec-1, Sigma-Aldrich, United States) and benzyloxycarbonyl-Val-Ala-Asp (zVAD, MP Biomedicals, Solon, OH, United States), which were dissolved in 10% dimethyl sulfoxide (DMSO, Sigma, United States) for subsequent use. zVAD and Nec-1 were used to specifically block cell apoptosis and necroptosis, respectively (Cui et al., 2016b; Ormsby et al., 2019). After 4 weeks of recovery after OVX, 48 rats in the OVX group were randomly assigned into four subgroups: OVX + vehicle group, OVX + zVAD group, OVX + Nec-1 group, and OVX + zVAD + Nec-1 group. Nec-1 (1.65 mg/kg/d) (Linkermann et al., 2012), zVAD (1.0 mg/kg/d) (Baumann et al., 2009), or an equal volume of 10% DMSO as the vehicle was injected intraperitoneally into the rats once per day for 4 weeks in the corresponding groups. The animals in the control group were administered with the vehicle solution. The rats were sacrificed 8 weeks after the operation by cervical dislocation because the peak time of osteocyte death was reported to be at 8 weeks after OVX (Cui et al., 2016a).

### Sample Harvest

Both the right and left tibial plateaus were dissected, and the soft tissue was removed from the bone. The bone marrow was rinsed out from the tibial plateau by repeated washing with PBS. Only the bone matrix of the tibial plateaus were stored in liquid nitrogen for subsequent western blot analysis (n = 6 rats per group). Some tibial plateaus were fixed in 10% neutral buffered formalin for 24 h for micro–computed tomography (micro-CT) analysis and histological analysis. The tibial plateaus used for the histological analysis were decalcified in 15% ethylenediaminetetraacetic acid (EDTA, pH 7.4) for 4 weeks and then subjected to the transferase dUTP nick end labeling (TUNEL) and immunohistochemistry reaction for detection of caspase-3. Other tibial plateaus were fixed in 2.5% glutaraldehyde for 24 h at 4°C and decalcified in 15% EDTA (pH 7.4) for 4 weeks for transmission electron microscopy (TEM).

### Micro–Computed Tomography

To analyze the bone microarchitecture, the tibial plateaus were scanned with micro-CT (viva CT40, SCANCO Medical AG, Zürich, Switzerland) using the following conditions: 15 µm resolution, 70 kVp, 114 μA, and 250 ms integration time. The volume of interest (29 × 29 × 29 µm^3^) was selected using a semiautomatic contouring method. 3D images were constructed using a series of planar transverse gray-value images. The microarchitectural parameters including bone mineral density (BMD), bone volume fraction (BV/TV), trabecular thickness (Tb.Th), trabecular number (Tb.N), and trabecular separation were calculated automatically using the micro-CT software.

### Western Blotting

The tibial plateaus frozen in liquid nitrogen were pulverized and homogenized in ice-cold buffer (Beyotime, Nantong, Jiangsu, China). The total protein concentration was determined with the bicinchoninic acid protein assay kit (Beyotime, Nantong, Jiangsu, China) according to the operating manual. The protein levels were determined by measuring the absorbance at 280 nm. Approximately 80 µg of protein was separated by SDS-PAGE and transferred to the polyvinylidene difluoride membrane (EMD Millipore, United States), which was then blocked with 5% nonfat dry milk solution for 1.5 h at room temperature and incubated overnight at 4°C with the following primary antibodies: anti-RIPK3 polyclonal antibody (ab152130, Abcam, Inc., Cambridge, MA, United States, 1:1000 dilution), anti-cleaved caspase-3 polyclonal antibody (ab2302, Abcam, Inc., Cambridge, MA, United States, 1:1000 dilution), anti-TNF-α monoclonal antibody (ab205587, Abcam, Cambridge, MA, United States, 1:1000 dilution), or anti-β-actin monoclonal antibody (sc-47778, Santa Cruz Biotechnology, CA, United States, 1:1000 dilution). Western blotting was performed routinely as previously described (Cui et al., 2016b). The protein bands of interest were visualized using an external cavity laser detection kit (Beyotime, Nantong, Jiangsu, China). The protein expression was quantitated by densitometry using Image Lab version 2.1 (Bio-Rad) and quantitative densitometric values for each kind of protein were normalized to β-actin.

### Immunofluorescence and *In Situ* Fluorescence TUNEL Staining

The bone matrix of the tibial plateaus was sliced into 4-µm sections for immunofluorescence and *in situ* fluorescence TUNEL staining. After three washes with 0.1 M PBS, the sections were incubated with 20 μg/ml proteinase K at 37°C for 15 min, treated with 0.1% Triton X-100, blocked with 10% goat serum, and incubated with polyclonal antibody against cleaved caspase-3 (Cell Signaling Technologies, Danvers, MA, United States; 1:100 dilution) overnight at 4°C, followed by anti-mouse IgG (EarthOx, San Francisco, United States). After three washes with PBS, the sections were analyzed by using the *in situ* cell death detection kit (Roche, Basel, Switzerland) following the manufacturer's protocol for the TUNEL method. All the sections were counterstained with 4′,6-diamidino-2-phenylindole (DAPI). Finally, the number of total cells and TUNEL-positive cells with immunostaining after caspase-3 (with and without cleaved caspase-3 immunostaining) were counted in three to five noncontiguous high-power fields (×200 magnification) for each specimen under a laser scanning confocal microscope (Leica TCS SP2, Wetzlar, Germany). The percentage and number of TUNEL-positive and cleaved caspase-3–positive cells were calculated by an experienced pathologist who was blinded to the experimental conditions.

### Transmission Electron Microscopy

The tibial plateau (1 mm^3^) was fixed in 2.5% glutaraldehyde for 24 h at 4°C, washed with 0.1 M PBS (pH 7.4), and demineralized in 15% EDTA for 4 weeks at room temperature. After three washes with PBS, tissue fragments were postfixed in 2% osmium tetroxide for 1 h, block-stained with 2% uranyl acetate, embedded in epoxy resin, and sliced into 80-nm sections which were subsequently stained with uranyl acetate and lead citrate and finally observed for the ultrastructures by using a transmission electron microscope (Hitachi-7500, Hitachi, Tokyo, Japan).

### Cell Culture and Treatments

Murine long bone osteocyte Y4 (MLO-Y4, 2.5×10^4^ cells, Cell Bank of the Chinese Academy of Sciences, Beijing, China) were plated in 12-well plates and cultured in DMEM/F12 (Gibco Life Technologies, Carlsbad, CA, United States) supplemented with 10% fetal bovine serum (FBS; Gibco Life Technologies, Carlsbad, CA, United States) in a humidified incubator containing 5% CO_2_ at 37°C. The cells were pretreated with DMSO (1%), Nec-1 (30 mmol/L), zVAD (25 mmol/L), or Nec-1 (30 mmol/L) + zVAD (25 mmol/L) for 30 min at 37°C, and then treated with TNF-α (100 ng/ml) for 24 h (Zhu et al., 2016). Flow cytometric analysis was performed using a FITC Annexin V apoptosis detection kit (BD Biosciences, San Diego, CA) according to the manufacturer's protocol. The cells were harvested, washed twice with PBS, and resuspended in 100 µL binding buffer. Cells were incubated for 15 min at room temperature in the dark after adding 5 µL FITC Annexin V and propidium iodide (PI), and then diluted with 400 µL of binding buffer. The cells were analyzed by flow cytometry (BD Biosciences, San Jose, CA). The cells in the four different quadrants were analyzed and interpreted as Q1: necrosis (Annexin V−/PI+), Q2: late apoptosis (Annexin V+/PI+), Q3: early apoptosis (Annexin V+/PI−), and Q4: viable cells (Annexin V−/PI−) (Malik et al., 2020). The percentages of late apoptosis plus necrosis and early apoptosis were calculated. Then, the cells (5×10^6^) were harvested and prefixed with 2.5% glutaraldehyde for 2 h. These cells were observed by TEM as described previously. The percentage of dead cells was calculated in six noncontiguous fields (×6,000 magnification) in each group.

### Statistical Analysis

Data were presented as mean ± standard deviation (SD). Statistical analysis was performed using SPSS software version 22.0. The significant differences between groups were evaluated using ANOVA followed by the least significant difference or Dunnett's post hoc test. The *p* value of <0.05 was considered statistically significant.

## Results

### Bone Loss in OVX Rats

**FIGURE 1:**
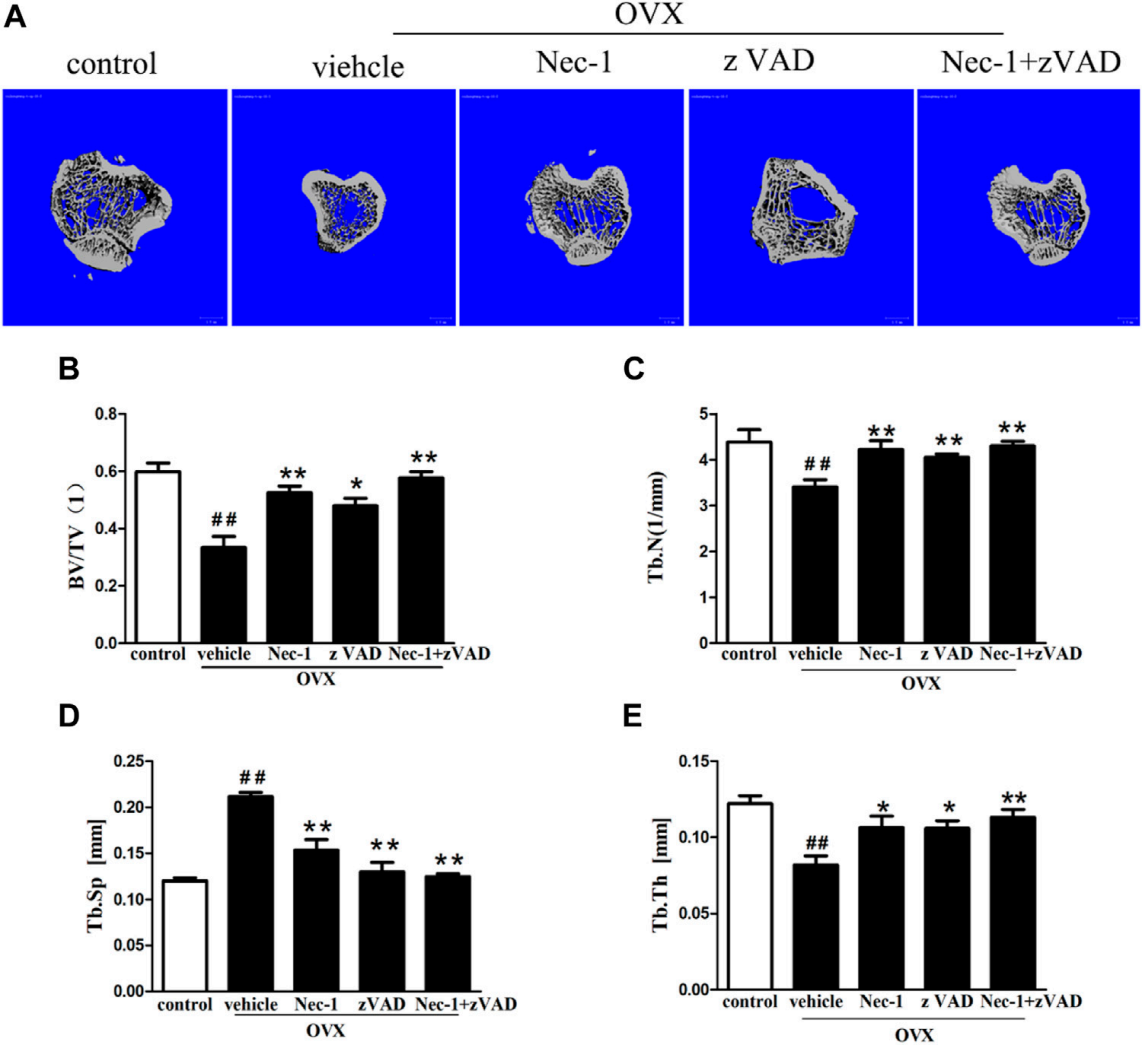
Bone loss was measured by micro-CT in OVX rats. The bone matrix of the tibial plateau was scanned using micro-CT, and the microarchitectural parameters (BV/TV, Tb.Th, Tb.Sp, and Tb.N) were calculated automatically. **(A)** Representative 2D reconstructed images were obtained in each group (scale bar = 100 μm) **(B)** BV/TV, **(C)** Tb.Th, **(D)** Tb.Sp, and **(E)** Tb.N were compared (n = 4). ##*p* < 0.01 compared with the control group, *p < 0.05 compared with the vehicle group, **p < 0.01 compared with the vehicle group.

About 8 weeks after OVX in SD rats, bone microarchitectures were measured by micro-CT (Figure 1). Figure 1A demonstrates the 2D reconstruction of the tibial plateaus and the BV/TV (Figure 1B), Tb.N (Figure 1C), Tb.Sp (Figure 1D), and Tb.Th (Figure 1E) in each group were compared. Compared to that in the control group, OVX led to significantly decreased BV/TV (Figure 1B), Tb.N (Figure 1C), and Tb.Th (Figure 1E) and increased Tb.Sp (Figure 1D), confirming that OVX resulted in substantial loss of bone mass and osteoporosis (*p* < 0.05). Compared with the vehicle group, BV/TV (Figure 1B), Tb.N (Figure 1C), and Tb.Th (Figure 1E) were remarkably increased in the Nec-1 group, zVAD group, and Nec-1 + zVAD group (*p* < 0.05), accompanied by reduced Tb.Sp (Figure 1D). In terms of BV/TV, the Nec-1 group, zVAD group, and Nec-1 + zVAD group led to approximately 57% increase, 44% increase, and 73% increase than vehicle group, respectively (Figure 1B). These three groups were also associated with about 24% increase, 19% increase, and 26% increase in Tb.N relative to the vehicle group (Figure 1C). For Tb.Sp, we found approximately 27% decrease in Nec-1 group, 39% decrease in zVAD group, and 41% decrease in the Nec-1 + zVAD group compared to the vehicle group (Figure 1D). Regarding Tb.Th, these three groups results in about 30% increase, 29% increase, and 40% increase than vehicle group (Figure 1E). These results suggested that treatment with Nec-1, zVAD and especially Nec-1 + zVAD could improve bone mass as compared to the vehicle group.

### Dead Osteocytes by TUNEL Staining

**FIGURE 2:**
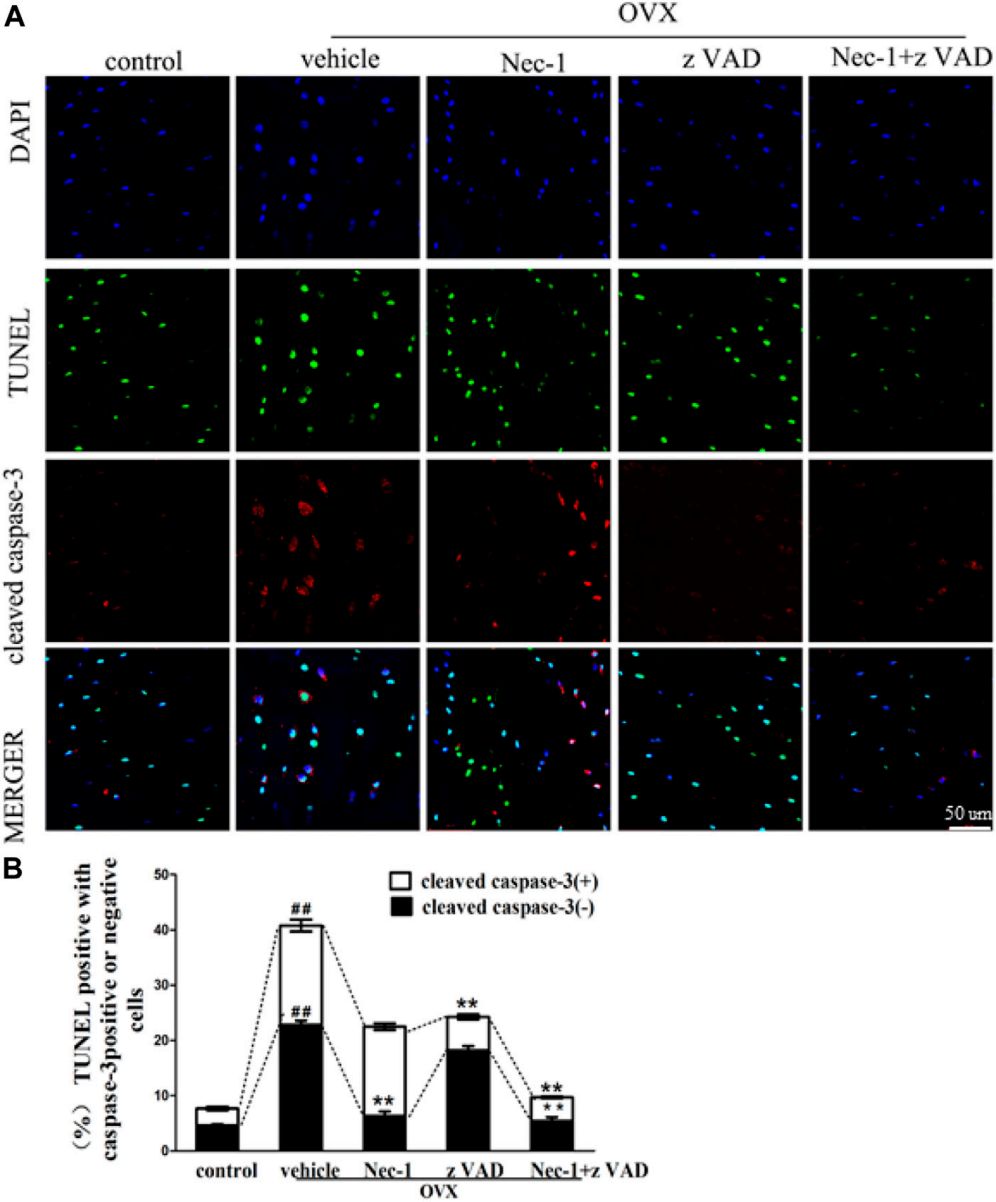
Osteocyte death was analyzed by the TUNEL method. **(A)** Immunofluorescence analysis of cleaved caspase-3 (red), *in situ* fluorescence TUNEL (green), and DAPI (blue) staining were performed in the bone matrix of the tibial plateau pretreated for 30 min with DMSO (1%), Nec-1 (30 mmol/L), zVAD (25 mmol/L), or Nec-1 (30 mmol/L) + zVAD (25 mmol/L) (scale bar, 50 μm). **(B)** Quantification of TUNEL-positive cells with or without cleaved caspase-3 was conducted in the bone matrix of the tibial plateau (n = 6). ^##^ *p* < 0.01 compared with the control group, *p < 0.05 compared with the vehicle group, **p < 0.01 compared with the vehicle group.

DNA fragments produced by dead cells in the bone matrix of the tibial plateau were detected by the TUNEL assay (Figure 2A). The percentage of TUNEL-positive cells in the bone matrix of the tibial plateau in the vehicle group was greater than in the control group (Figure 2B). The percentage of TUNEL-positive cells was reduced significantly after treatment with zVAD or Nec-1 alone, and the significance of reduction became more robust in the Nec-1 + zVAD group. It was important to emphasize that the number of TUNEL-positive cells in OVX rats treated with Nec-1 or Nec-1 + zVAD was significantly lower than in rats treated with zVAD alone (Figures 2A,B). Furthermore, anti-active caspase-3 staining was conducted to identify cell apoptosis (Günther et al., 2011; Liedtke et al., 2011; Vanlangenakker et al., 2012). These results indicated that both necroptosis and apoptosis led to the death and loss of osteocytes, and cell necroptosis had a bigger role in the loss of osteocytes than cell apoptosis.

### Morphological Observation of Osteocytes by TEM

**FIGURE 3:**
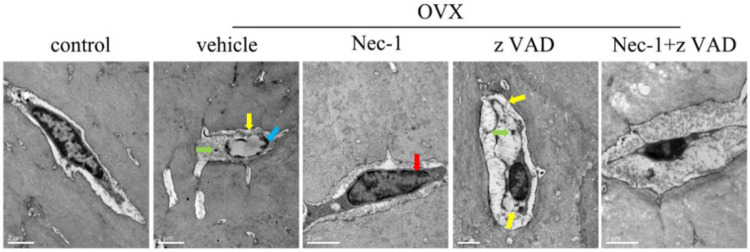
Dead osteocytes were seen by TEM. (A) TEM images of osteocytes in sham-operated rats and OVX rats treated with vehicle, Nec-1, zVAD, or Nec-1 + zVAD (scale bar = 2 μm). Red arrow: condensed nucleus, blue arrow: chromatin clumping, green arrow: swelling organelle, yellow arrow: membrane rupture. ##*p* < 0.01 compared with the control group, *p < 0.05 compared with the vehicle group, **p < 0.01 compared with the vehicle group.

To further examine the morphological characteristics of dead osteocytes, we observed the ultrastructures of osteocytes in the bone matrix of the tibial plateaus using TEM at 8 weeks after OVX. The typical morphological features of necroptotic osteocytes included swelling of the organelles, rupture of the plasma membrane, chromatin clumping, and intracellular vacuoles, which were seen in the vehicle group and zVAD group. Nuclear condensation of apoptotic cells was observed in the Nec-1 group (Figure 3). These indicated that osteocyte death was mainly caused by necroptosis rather than apoptosis at 8 weeks after OVX.

### RIPK3 and Caspase-3 Expression

**FIGURE 4:**
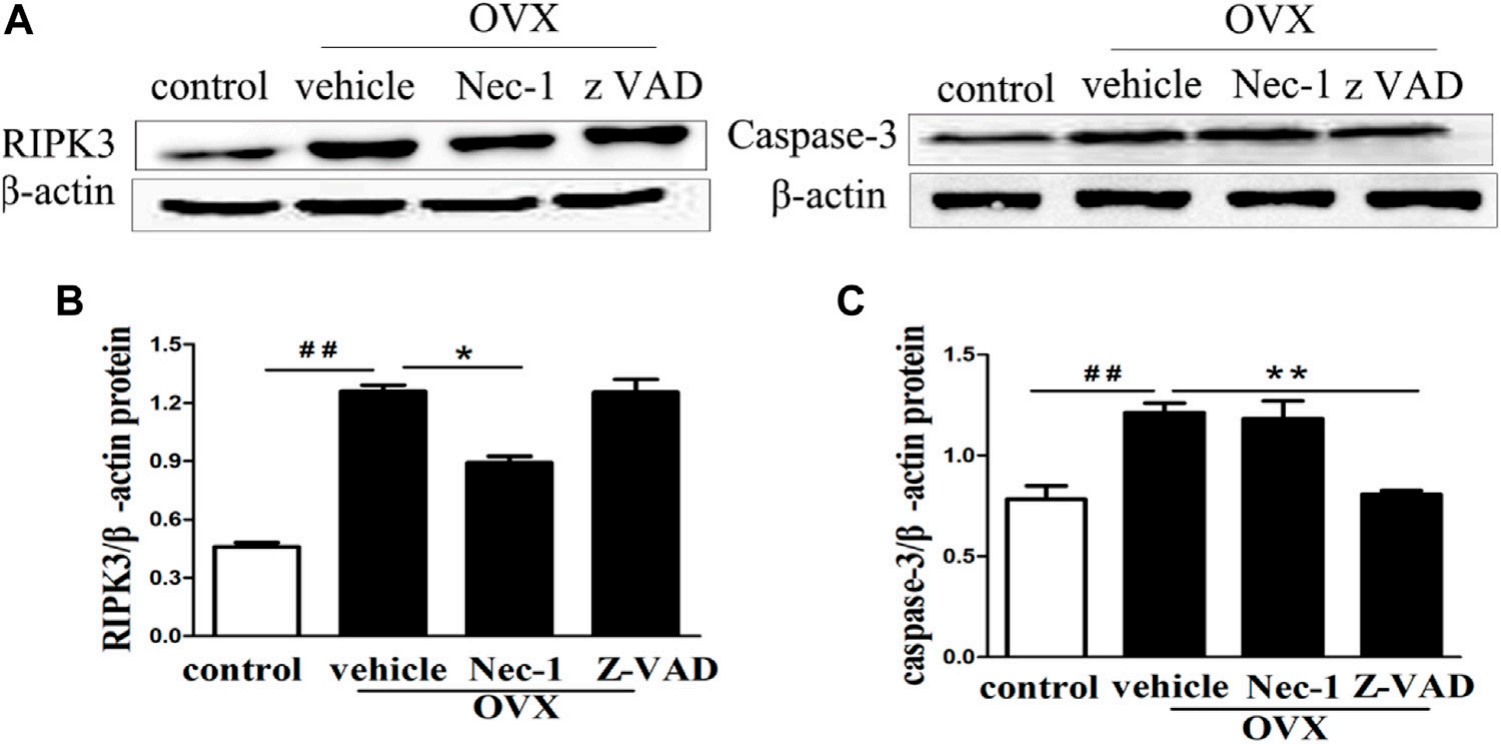
**(A)** Western blot of RIPK3 and caspase-3 protein expression in the osteocytes of each group. The quantitative analyses of **(B)** RIPK3 and **(C)** caspase-3 protein expression. Nec-1 and zVAD were associated with significantly reduced expression of RIPK3 and cleaved Caspase-3 (n = 6), respectively. ^##^ *p* < 0.01 compared with control group, *p < 0.05 compared with the vehicle group, **p < 0.01 compared with vehicle group.

RIPK3 and cleaved caspase-3 were the important markers to identify necroptosis and apoptosis of cells, respectively. Their protein expression levels were measured in the bone matrix of OVX rats by using western blotting. The results showed that RIPK3 and caspase-3 protein levels were increased in the bone matrix of OVX rats compared with those in sham-operation rats (Figure 4A). Treatment with Nec-1 significantly reduced the RIPK3 level (Figure 4B), while the caspase-3 level was substantially decreased in the zVAD group than in the Nec-1 group (Figure 4C). This confirmed that both necroptosis and apoptosis contributed to the death and loss of osteocytes.

### TNF-α Production in the Bone Marrow of OVX Rats

**FIGURE 5:**
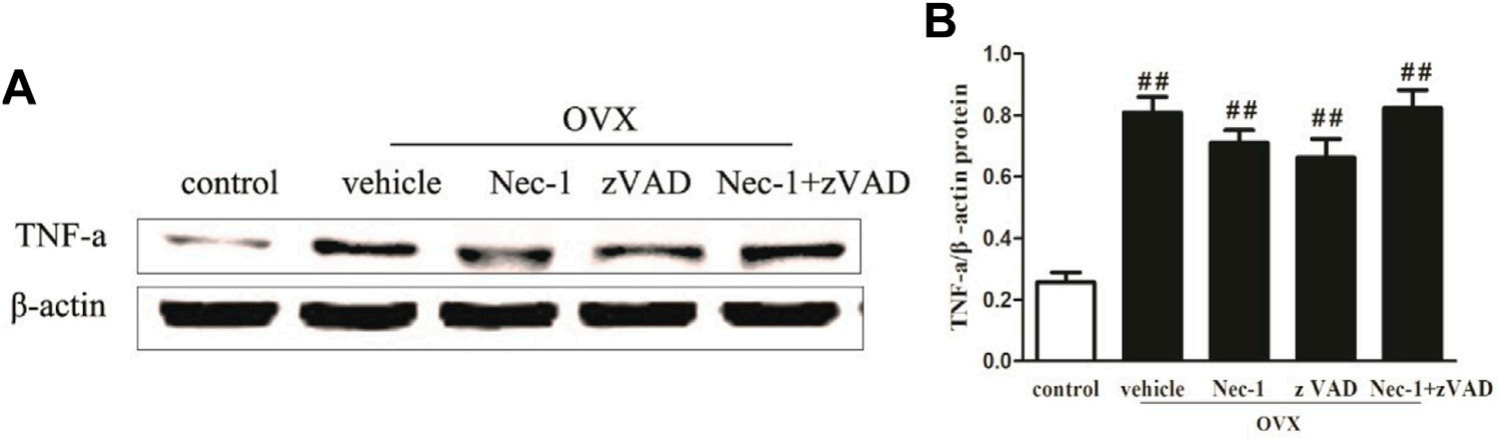
TNF-α expression in OVX rats. **(A)** Western blot of TNF-α protein expression and **(B)** its quantitative analysis in the osteocytes of sham-operated and OVX rats treated with vehicle, Nec-1, or zVAD (n = 6). ##*p* < 0.01 compared with the control group, *p < 0.05 compared with the vehicle group, **p < 0.01 compared with the vehicle group.

TNF-α is mainly produced in hematopoietic precursor cells and is one important inflammatory factor which induces PMOP (Liu et al., 2020; Lu et al., 2020). TNF-α could directly enhance the expression of the receptor activator of nuclear factor κΒ ligand (RANKL) in osteocytes and promote osteoclast formation (Kitaura et al., 2020). We detected the expression of TNF-α in the bone marrow of OVX rats in each group by western blotting and found that TNF-α expression was significantly lower in the control group and increased sharply in the vehicle group (Figures 5A,B). Although treatment with zVAD or Nec-1 showed a decreasing trend in TNF-α protein expression compared to the vehicle group, no statistical difference was seen between them.

### TNF-α–Induced Death of MLO-Y4 Cells

**FIGURE 6:**
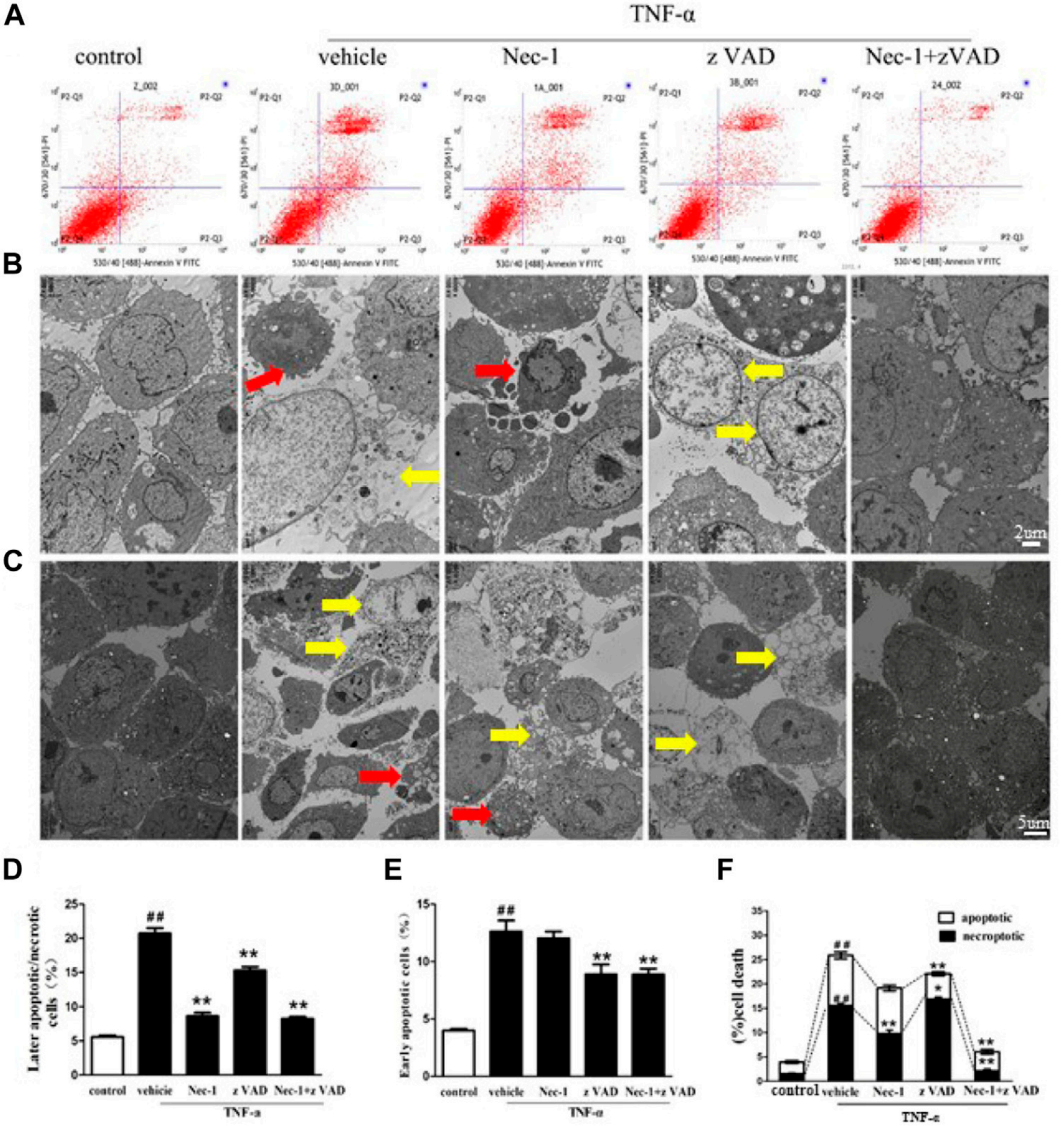
TNF-α–induced necroptosis and apoptosis of MLO-Y4 cells. **(A, D, E)** Necrotic and apoptotic osteocytes were tested using the flow cytometer. MLO-Y4 cells were pretreated for 30 min with DMSO (1%), Nec-1 (30 mmol/L), zVAD (25 mmol/L), or Nec-1 (30 mmol/L) + zVAD (25 mmol/L) and then treated with TNF-α (100 ng/ml) for 24 h *in vitro*. Then, MLO-Y4 (upper panel) cells were stained with FITC Annexin V and PI to determine necrotic and apoptotic osteocytes by flow cytometry. **(B, C, F)** TEM images of osteocytes pretreated for 30 min with DMSO (1%), Nec-1 (30 mmol/L), zVAD (25 mmol/L), or Nec-1 (30 mmol/L) + zVAD (25 mmol/L) and then treated with TNF-α (100 ng/ml) for 24 h *in vitro* (B: scale bar = 2µm, C: scale bar = 5 µm). Red arrow: apoptotic cells, yellow arrow: necroptotic cells. ##*p* < 0.01 compared with the control group, *p < 0.05 compared with the vehicle group, **p < 0.01 compared with the vehicle group.

Estrogen deficiency increased the expression of TNF-α in the bone marrow of OVX rats, and we explored the influence of TNF-α abnormality on the death of osteocytes. Therefore, we cultured MLO-Y4 cells in order to compare necroptosis with apoptosis for osteocyte death. Flow cytometry and TEM analyses revealed that the cell death rate of MLO-Y4 cells stimulated by TNF-α was significantly increased. Compared with the control group, early rate of cell death in the TNF-α group was significantly increased, and zVAD effectively reduced the early death rate of MLO-Y4 cells, but Nec-1 had no significant effect on the early death rate (Figures 6A,D). The late death rate in the TNF-α group was also significantly higher than in the control group. Both Nec-1 and zVAD remarkably reduced late necrosis and apoptosis of MLO-Y4 cells, and Nec-1 could result in a significantly lower rate of cell death than did zVAD (Figures 6A,E). By contrast, the incidence of necroptotic cells was greater than that of apoptotic MLO-Y4 cells in response to TNF-α stimulation (Figure 6B, C, F). TNF-α combined with zVAD significantly decreased the rate of apoptosis but increased the incidence of necroptosis. Furthermore, TNF-α combined with Nec-1 only blocked necroptosis but did not change the rate of apoptosis (Figure 6B, C, F). In addition, Nec-1 plus zVAD sharply reduced the incidence of apoptotic and necroptotic MLO-Y4 cells compared to vehicle intervention. These results confirmed that the death of osteocytes after TNF-α stimulus was caused by necroptosis and apoptosis, and cell necroptosis had a bigger role in the loss of osteocytes than did cell apoptosis. These findings are consistent with the results *in vivo*.

## Discussion

Death and loss of osteocytes display crucial roles in the incidence of PMOP, and osteocyte apoptosis has been widely accepted as an important pathological process in PMOP (Tomkinson et al., 1998; Emerton et al., 2010). Surprisingly, our previous study found that osteocyte necroptosis was also responsible for osteocyte loss in OVX-induced osteoporosis (Cui et al., 2016a). Both apoptotic and necroptotic cell deaths are regarded as two important approaches to cause excessive loss of osteocytes (Cui et al., 2016a), but their roles in the loss of osteocytes and PMOP are not well compared. The clarification of this problem would greatly benefit developing preventive and therapeutic strategies for PMOP. Our results confirm that both necroptosis and apoptosis contribute to the excessive loss of osteocytes and bone loss in OVX-induced osteoporosis *in vivo* and *in vitro*, and furthermore, necroptosis may produce a greater influence on the death of osteocytes than apoptosis at 8 weeks after OVX.

It is difficult to distinguish necroptotic cells and apoptotic cells due to the absence of specific cellular markers. We used several methods to determine necroptotic death of osteocytes *in vivo* and *in vitro*. TEM was used to illustrate the morphological and ultrastructure features of apoptotic and necrotic osteocytes (Galluzzi et al., 2009). The typical features of necroptotic cell osteocytes included the expansion of osteocyte volume, swelling of the organelles, rupture of the plasma membrane, loss of organelle contents, and extensive formation of intracellular vacuoles in the bone matrix of OVX rats, especially in the vehicle and zVAD groups. By contrast, only vehicle intervention was associated with cell shrinkage and nuclear condensation that were typical in apoptotic cells. Furthermore, treatment with Nec-1 improved these ultra-microstructural changes. These findings confirm that necroptotic cell death was crucial for osteocyte loss. More interestingly, significantly fewer apoptotic osteocytes were observed by TEM than necrotic osteocytes. These indicated that necroptosis may produce a greater impact on the death of osteocytes than apoptosis in OVX-induced osteoporosis. The osteocytes are surrounded by the mineralized matrix and the apoptotic osteocytes are not rapidly engulfed by neighboring cells such as osteoclasts and macrophages (Cerri et al., 2000; Boabaid et al., 2001; Florencio-Silva et al., 2015), which indicates that the death of osteocytes is mainly caused by secondary necrosis and this supports our finding.

The TUNEL method was regarded as the gold standard to identify cell death, including necroptosis and apoptosis, both of which could generate DNA fragments and react with TUNEL staining mixtures (Trichonas et al., 2010). We used the TUNEL assay to detect dying cells in the bone matrix of OVX-induced rats. The results demonstrated that the number of TUNEL-positive cells and their percentage in the vehicle group were both increased. In addition, treatment with Nec-1 significantly reduced the number and percentage of TUNEL-positive cells than did zVAD. To further discriminate the types of TUNEL-positive cells observed, anti-active caspase-3 immunostaining was performed. Typically, TUNEL-positive cells and no immunoreactivity to the active caspase-3 can be considered as necroptotic cells, while TUNEL-positive cells that exhibited positivity to the active caspase-3 are characterized as apoptotic cells (Günther et al., 2011; Liedtke et al., 2011; Vanlangenakker et al., 2012). These methods also confirmed that these two types of cells were both significantly reduced in Nec-1 and zVAD intervention, but Nec-1–induced reduction appeared to be more obvious than zVAD-induced reduction.

To obtain more convincing evidence, we cultured MLO-Y4 cells *in vitro*, and cell death was observed by TEM and quantitatively analyzed. The results showed that the incidence of apoptosis and necroptosis was significantly elevated in MLO-Y4 cells stimulated with TNF-α. Importantly, we found that the incidence of necroptotic MLO-Y4 cells was significantly higher than that of apoptotic cells. These *in vitro* results were consistent with the results of OVX-induced rats. These results demonstrated that necroptosis had greater roles in osteocyte death than did apoptosis. However, these findings are not supported in the microarchitectural parameters, suggesting that apoptosis and necroptosis of osteocytes is just one important factor to affect bone loss and osteoporosis. Other factors including osteoclastic resorption and bone formation by osteogenic differentiation of the bone marrow mesenchymal stem cells also had valuable roles in bone loss and osteoporosis (Li et al., 2019; Wang et al., 2020; Zheng et al., 2021).

RIPK3 is a key switch molecule between the necroptotic and apoptotic pathways and is also recruited to the necrosome complex through direct interactions between RIP homotypic interaction motif domains of RIPK1 and RIPK3 (Galluzzi et al., 2009; Günther et al., 2011). (Linkermann et al., 2012) reported its functional predominance of necroptosis over apoptosis in renal ischemic/reperfusion injury. Our study demonstrated that the protein level of RIPK3 was remarkably increased in the bone matrix of OVX rats, and this increase could be significantly blocked by Nec-1 but not zVAD, suggesting that RIPK3 mainly regulated necroptotic cell death rather than apoptotic death. Additionally, if apoptotic osteocytes are not immediately cleared by macrophages, secondary necrosis occurs with cytoplasmic membrane ruptures. These stimulate the generation of multiple immunostimulatory cytokines and proinflammatory molecules, the aggregation and energization of immune cells (e.g., macrophages, monocytes, and neutrophils), which also induce the secretion of RANKL and bone resorption (Komori, 2013; Komori, 2016).

We also should consider several limitations. Firstly, we only used the bone matrix of the tibial plateaus after flushing out the medullary cavity during the extraction of protein. The harvested tibial plateaus used for western blotting not only contained mainly osteoblasts but also comprised other cells such as osteoclasts, which may affect the results of western blotting. Secondly, our results found that necroptosis may have a greater influence on the death of osteocytes than apoptosis at 8 weeks after OVX surgery, but only one follow-up time was included in our study. Necroptosis and apoptosis displayed different roles in the death of osteocytes at various time points. For instance, apoptosis appeared to be the most prevalent type of cell death in osteocytes within 14 days after OVX surgery, while the necroptosis had improved function to cause osteocyte death in the following weeks (Emerton et al., 2010; Cui et al., 2016b). Thirdly, necroptosis showed greater roles in osteocyte death than apoptosis at 8 weeks after OVX surgery, which was not consistent with the microarchitectural parameters of micro-CT in OVX rats, indicating that other factors such as osteoclastic resorption and osteogenic differentiation also affected bone mass and osteoporosis.

## Conclusion

Both necroptosis and apoptosis are confirmed to result in the death and excessive loss of osteocytes in OVX-induced osteoporosis, and necroptosis may generate a greater impact on the loss of osteocytes than apoptosis.

## Data Availability

The original contributions presented in the study are included in the article/Supplementary Material; further inquiries can be directed to the corresponding author.
